# Intracutaneous Injections of Sterile Water over the Secrum for Labour Analgesia

**Published:** 2009-04

**Authors:** Kirti N Saxena, Hitesh Nischal, S Batra

**Affiliations:** 1 Professor, Department of Anesthesiology and Intensive care, Maulana Azad Medical College & associated Lok Nayak Hospital, New Delhi; 2 Senior Resident, Department of Anesthesiology and Intensive care, Maulana Azad Medical College & associated Lok Nayak Hospital, New Delhi; 3 Director-Professor & Head, Department of Obstetrics & Gynaecology, Maulana Azad Medical College & associated Lok Nayak Hospital, New Delhi

**Keywords:** Labour analgesia, Sterile water injections

## Abstract

**Summary:**

During first stage of labour, many women suffer from lower back pain. Since cutaneous afferents from the lower back converge to the dorsal horns in the same segments there is anatomical support for the lower back pain being a referred pain. Intracutaneous injections of sterile water in the skin over the sacrum have been shown to relieve the pain of labour and it is free from negative side effects associated with use of other methods.

The study was conducted after approval of institutional ethical committee on 100 pregnant patients admitted to the labour room of Lok Nayak hospital, New Delhi. Patients received 4 intracutaneous injections of sterile water or normal saline 0.5ml in the lumbo-sacral region. Pain scores, progress of labour and fetal outcome were studied. There was significant reduction of pain scores in the sterile water group but not in the normal saline group at 10, 45 and 90 minutes after injection. There was no difference in the progress of labour and fetal outcome between the two groups. To conclude intracutoneous sterile water injections over the sacrum is a simple and effective method to control pain during labour.

## Introduction

Labour pain is a unique visceral pain associated with a wonderful and meaningful life event – the birth of a baby. From many years many methods have been used to make labour smooth and pain free. Many are effective but none proved to be free from side effects. Our aim was to discover a method which will be not only effective but also free from side effects.

During first stage of labour, many women suffer from lower back pain. The cervix and corpus uteri are supplied by afferent neurons ending in the dorsal horns of spinal segments T10-L1^1^. Since cutaneous afferents from the lower back converge to the dorsal horns in the same segments there is anatomical support for the lower back pain being a referred pain^2^. Using gate control theory or counter irritation theory, stimulation of specific areas can relieve a referred pain^3^. The mechanism has been described as counter irritation, a process by which localized pain felt in one part of the body may be relieved by irritating the skin in same dermatomal distribution with either a hot, cold, scratchy, or electrical stimulus. The sterile water injections are thought to cause distension in the skin, which stimulates nociceptors and mechanoreceptors.

Intracutaneous injections of sterile water in the skin over the sacrum have been shown to relieve the pain of labour in open studies and in controlled prospective studies^4^–^6^. The method has also been used to treat acute attacks of urolithiasis^7^, neck and shoulder pain^8^ and the chronic myofascial pain syndromes^9^. This method was found to be simple and efficient. No side effects have been observed other than injection site burning pain lasting for few seconds in previous studies and it is free from negative side effects associated with use of other methods like epidural analgesia and intramuscular narcotics^4^,^9^.

The aim of the present study was to discover whether intracutaneous sterile water injections are effective to relieve back pain during labour and free from side effects in Indian parturients. The study was carried out by randomized, double-blinded trial including a placebo treated patient group with normal saline and comparing with sterile water injections treated patients group.

## Methods

The study was conducted after approval of institutional ethical committee comprising of 100 pregnant patients admitted to the labour room of Lok Nayak hospital, New Delhi. To qualify for entry into the trial they had to be in the first stage of labor (cervical dilatation around 4 cm) and require pain relief of lower back pain on admission or during their stay in the labour room. Written informed consent was taken by all the patients.

Selection criteria- All patients in first stage of labour

Exclusion criteria-1. Infection in the area of injection

2. Patients not willing for the procedure

3. Patients who have received any analgesic following onset of labour

The women were randomized into 2 groups by computer generated numbers.

**Fig 1:**
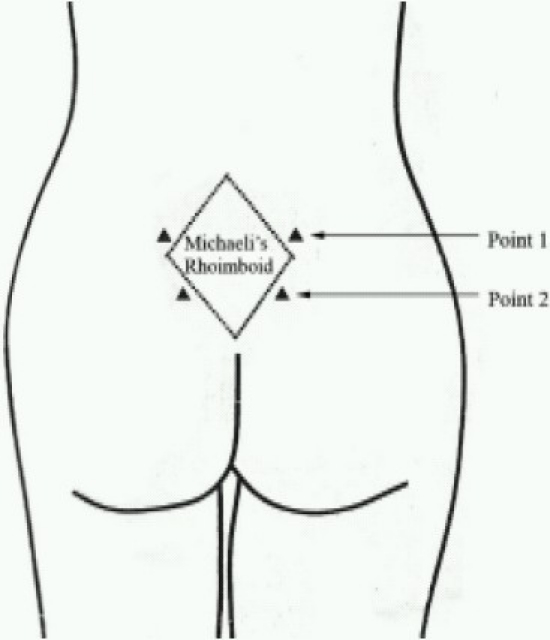
The Michaelis' rhomboid and points for injection (From: Martensson L, Wallin. G. Labour pain treated with cutaneous injections of sterile water: a randomized controlled trial. Br J Obstet Gynaecol,1999;106: 633-637).

Sterile water injection group patients received 4 intracutaneous injections of 0.5 ml sterile water in the lumbar- sacral region in the sitting position. One injection was given at the posterior superior iliac spine(Point.1) on both sides and second injection at 1 cm medial, and 1-2 cm inferior to the first point on both the sides (Point.2) using an insulin needle. These points overlie the area called Michaelis' rhomboid (Fig 1)

Normal saline injection group patients received injections of 0.5 ml isotonic saline in the same region using an insulin needle.

All the solutions were prepared by the first author while the second anaesthesiologist and the obstetrician were blinded to solution used. All the injections were given by the two anaesthesiologists simultaneously on each side to coincide with the pain of labour contractions. All the patients had a brief stinging pain when the injection was given. The pain lasted longer in the serile water group but subsided within a few seconds.

The following parameters were recorded

- Pain assessment with the help of verbal numerical rating scale, 0-100 at 10min, 45min and 90min after giving the injections was carried out by the second anaesthesiologist who was blinded to the solution injected
- Physician assessment of pain (performed by obstetric senior resident on duty at that time in labour room), at 10min, 45min and 90min after giving the injections. The pain relief was graded as none, mild, moderate and excellent. The obstetrician was blinded to the solution injected for pain relief
- Progress and duration of labour as assessed by the obstetrician
- Operative intervention
- Apgar score of the neonate

Total number of women investigated was 100, 50 in each group. The sample size calculated to achieve a significant end point of pain score of 100 to pain relief pain score of 30 was 6 in each group to achieve a power of 80%. The sample size of 6 was derived from the hypothesis that pain would be reduced from 100 to 30 on the VAS scale. Statistical analysis was done by using T- test for equality of means of patient age and gestational age and also for Apgar score, Chi-square tests for parity, cervical dilatation, physician assessment of pain. Mann-Whitney U and Wilcoxon W tests respectively were done for VAS at 0, 10, 45, 90minutes (between groups) and duration of labour and for statistical significance of change of VAS from 0 to 10 min, 0 to 45 min and 0 to 90 min in each group.

## Results

The women in the 2 groups were similar with regard to age, parity and gestational age (Table 1). Comparing the cervical dilatation (Table 1) between groups at the beginning of the study was also statistically insignificant (p=1). All patients were in active stage of labour.

**Table 1:** Demographic and clinical data before treatment(mean±SD, n)

	Sterile water group	Normal saline group
Age (years)	24.72±3.61	25.80±3.64
Parity(primi/multi)	25/25	20/30
Gestational age (weeks)	38.12±1.38	37.72±1.08
Cervical dilatation(cm)	3.91±0.54	3.8±0.56

**Figure 2:**
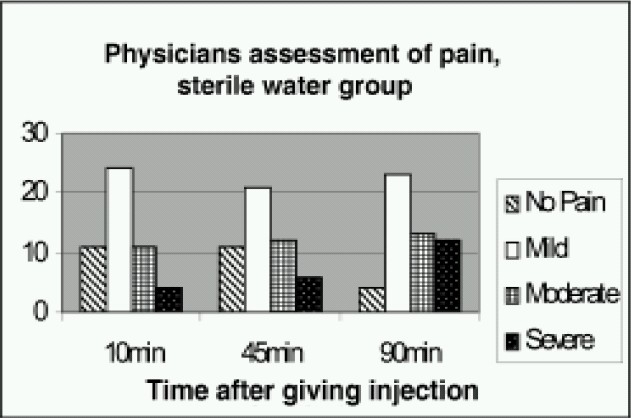
Physicians assessment of pain at different times after giving the sterile water injections

**Figure 3:**
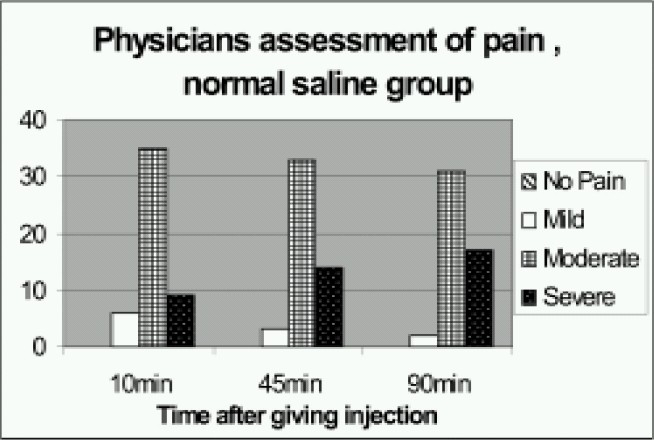
Physicians assessment of pain at different times after giving the normal saline injections

The mean VAS score at start of treatment was 75.3 in sterile water group and 74.7 in normal saline group with statistical insignificance between both groups. The mean VAS pain score 10 minutes after treatment when compared to the pretreatment score was found to be reduced (statistically highly significant) in sterile water group but not in normal saline group. Mean VAS pain score at 45min and 90min was also found to be reduced (Table 2) considerably in the sterile water group but not the saline group. There was highly significant reduction of VAS scores at 10, 45, 90min compared to VAS at 0min (p<0.005) in the sterile water group (Table 3). Similar changes were observed in physician assessment of pain at 10, 45, and 90 minutes (Fig 2,3). There was significant difference (p<0.05) between the two groups at all three times. Many patients in the sterile water group went off to sleep after receiving the injections.

**Table 2:** Mean VAS scores at different times

Group	VAS at 0min	VAS at 10min	VAS at 45min	VAS at 90min
Sterile Water	75.3±23.04	34.2±28.70	33.2±32.67	49.3±33.96
Normal saline	74.7±23.45	73.4±23.48	77.4±20.78	83.7±18.81
p value-sterile water vs normal saline	0.29	0.00	0.00	0.00

**Table 3:** Statistical significance in change in VAS score in ‘Sterile water group’

	From 0 to 10min	From 0 to 45min	From 0 to 90min
p value	0.000	0.000	0.000

No other method of pain relief was used in both the groups other than using sterile water or placebo with normal saline. As per the labour room protocol of our hospital, uncomplicated parturients are not given labour analgesia unless they demand it. In 8 out of 50 sterile water group, crowning of the baby's head was detected in early stage by physician with absence of complaint of pain by the patient.

The mean period between injections and delivery was 4.01±2.15 hrs in sterile water group and 4.17±2.30 hrs in normal saline group. The difference was not significant. Only two patients in sterile water group underwent operative delivery i, e. lower segment caesarean section(LSCS) the indications being cephalopelvic disproportion and previous LSCS.

Mean Apgar score of the newborns in both the groups was 8.7±0.5 and 8.58±0.15 respectively. There was no difference between the two groups.

## Discussion

Uterine contractions are felt as back pain because rami of T10 – L1 supplying the uterus also supply the skin over the lumbo-sacral area^1^. The cutaneous branches of the lumbar and the lower thoracic nerves cover a considerable caudal area. They transmit referred pain from uterus to a skin area over the vertebrae L3-S2^1^. The injections were given adjacent to the Michaelis' rhomboid because this is the area where referred pain from the uterine contractions were felt.

Injecting solutions of osmolality other than blood irritates biological tissues. Sterile water injection evokes intense pain, probably due to the difference in osmolality^10^. Stimulation of skin during administration of sterile water gives rise to a similar gate control effect and /or a stimulation of the endogenous opioid system as TENS and acupuncture do. Acupuncture for analgesia purposes can be applied to specific traditional points following meridians. The points are often located far from the painful area. However, needling at sites segmentally related to the painful site may be equally or even more effective^11^. The intracutaneous injections of sterile water may act as a long lasting segmental acupuncture^4^. In the clinically controlled double blind study by Bengtsson et al^7^, acute ureteric colic was treated by injecting four papules of sterile water over the cutaneous area where projected pain from the kidney and the upper urinary tract was felt. Because sterile water is hyposmolar, it probably irritates the nerves leading to brief pain initially which is followed by analgesia in the same way as acupuncture while saline being isosmolar with blood does not irritate the nerves at all and therefore does not lead to analgesia.^10^

Epidural analgesia using opioids is the most potent method for women in labour in need of effective analgesia^12^. Intramuscular administration of narcotics can also reduce the pain of labour pain but this method is limited by negative side effects such as maternal drowsiness, nausea and vomiting as well as neonatal respiratory depression^13^. Because of the risk of losing control^14^ or potential negative effects on baby^13^, many women do not want pain relief with narcotic drugs. In India, due to paucity of anaesthesiologists, epidural analgesia is not used frequently for labour in most government hospitals. Also, most women expect labour to be painful and accept it as a reality. The sterile water group patients were visibly happier after receiving the injections. Injection of sterile water in the skin over the sacrum in the points along the Michaelis' rhomboid has been used with success by various authors^4^–^6^,^9^. These authors used 0.1 ml volume while we used 0.5 ml because of the contention that it is very difficult to pin-point the exact point of injection which we tried to overcome with a higher volume. We observed that patients had less pain during the second stage also but we did not try to evaluate as it was beyond the scope of our protocol. In our study no attempts were made to define the mechanism of action more exactly. Because of non detection of crowning in few patients it was advised to monitor progress of labour more frequently in the absence of complaint of pain by patient. The role of sterile water injections in second stage of labour needs further evaluation.

This study is limited by the fact that the duration of the study of pain was restricted to 90 minutes only and the maximum duration of pain relief could not be studied. However, we had aimed only to study whether this method was actually as effective as quoted in various studies or not.

In summary, sterile water given intracutaneously seem to be an efficient and simple method for antagonizing parturition low back pain. It provides a powerful effect lasting from 45-90 minutes, and may be repeated during labour. It may be a very useful treatment for low back pain during the initial part of the first stage of labour even for those women that may require epidural analgesia when labour proceeds. It can easily be administered by the patient's midwife and no side effects have been observed other than a burning pain lasting for a few seconds
